# Improving the care pathway for women who request Caesarean section: an experience-based co-design study

**DOI:** 10.1186/s12884-016-1134-2

**Published:** 2016-11-09

**Authors:** Sara L. Kenyon, Nina Johns, Sandhya Duggal, Ruth Hewston, Nicola Gale

**Affiliations:** 1 Institute of Applied Health Research, University of Birmingham, Edgbaston, Birmingham, B15 2TT UK; 2 Birmingham Women’s NHS Foundation Trust, Mindelsohn Way, Birmingham, West Midlands B15 2TG UK; 3 Research Fellow, Institute of Applied Health Research, University of Birmingham, 255 Walmley Road, Walmley, Sutton Coldfield, West Midlands B76 2PN UK; 4 Health Services Management Centre, University of Birmingham, Park House, 40 Edgbaston Park Road, Edgbaston, Birmingham, B15 2RT UK

**Keywords:** Maternal request for Caesarean section, Experience based co-design, Qualitative methods, Interviews

## Abstract

**Background:**

Maternal request for Caesarean section is controversial and yet the NICE Caesarean section Guideline recommends that that if this is requested, following discussion of the risks and benefits, women should be supported in their choice. There was a desire to improve the pathway at Birmingham Women’s NHS Foundation Trust.

**Methods:**

Experience-based co-design methodology uses service user and clinicians experiences collected using qualitative methods to jointly re-design services. Firstly semi-structured interviews were conducted to elicit the views and experiences of health care professionals and women who requested Caesarean section (with and without medical indication). Analysis identified key themes arising from the health care professionals’ interviews and ‘touch points’ (key moments or events related to the experience of care) arising from the interviews with women.. Separate workshops were then held with each group to ensure these resonated and to identify key areas for service improvement. At the first joint workshop a pathway using ‘audio clips’ demonstrating women’s agreed ‘touch points’ prompted discussion and joint working began to change the pathway. A final second workshop was held to agree changes to the pathway.

**Results:**

Interviews were conducted with health care professionals (*n* = 22, 10 consultant obstetricians and 12 midwives) and women (*n* = 15). The women’s ‘touch points’ included repetition of request, delay in the decision for Caesarean section to be made, feeling judged, and that information was poor with similar findings identified from the health care professionals. Joint working resulted in a revised pathway for women who request Caesarean section*.*

Changes to the pathway for women as a result of the work include written information about ‘The way your baby may be born’ which is given to the woman followed by a discussion about mode of birth around the 16 week appointment. If the woman wishes to have a Caesarean section, referral is made to appropriate health care professionals (e.g., Consultant Midwife, counsellor) only if support and information would be useful. If Caesarean section is requested, woman is referred to a consultant obstetrician for an appointment at 20/40, with a decision by 28/40. Recording this in the notes minimises repeated challenge described by women. Final consent and timing of Caesarean section remain as recommended.

**Conclusion:**

This has resulted in changes to the pathway agreed by a co-design process and which are acceptable to both health care professionals and women. Use of such methodologies should be considered more frequently when implementing service change.

## Background

In England the rates of Caesarean section have risen from 9 % of births in 1980 to 25.4 % in 2013 (http://www.birthchoiceuk.com/Professionals/BirthChoiceUKFrame.htm?http://www.birthchoiceuk.com/Professionals/statistics.htm). Indications for the procedure vary, but one possible contributor to this rise may be an increase in maternal requests for Caesarean section [1], although the exact extent to which women request Caesarean section in the absence of clinical indications is not clear, with studies suggesting it varies from 0.3 to 14 % [2].

While it is widely accepted that matters such as place of birth, method of pain relief, position in labour or presence of a birth partner are accepted matters of maternal choice, there is controversy surrounding whether the woman should have the right to choose to have her baby by Caesarean section [3]. Reasons for this decision include fear of childbirth [4], avoidance of the pain of labour and of the risk of damage to the perineum, previous birth experiences [5] as well as convenience of a planned birth [6].

Recent evidence has suggested that, while support and control are important determinants of satisfaction with the birth experience [7, 8] fulfilment of the request for either Caesarean section or vaginal birth does not guarantee a positive birth experience [9]. A recent review [2] has found few studies that addressed women’s own perceptions of their role in decision making, which is perhaps surprising. There has been an increasing drive for better understanding of patient experience, and more meaningful patient involvement in service design and improvement [10], although efforts have sometimes been limited by failure to engage in depth with patients’ subjective experiences [11]. Experience Based Co-Design [12] utilises in depth accounts of experiences from service users to re-design services. The method has been used and developed in the healthcare setting over the last 10 years and is an approach to improving services that combines participatory design and user experience to bring about quality improvement. As such it provides an established research methodology for enabling Trusts to fulfil their statutory duties and involve patients and the public in improving services (https://www.england.nhs.uk/wp-content/uploads/2013/09/trans-part-hc-guid1.pdf). In-depth interviews are used to elicit the views of both health care professionals and service users. This methodology has been used successfully in a variety of health care settings including emergency departments [13], breast and lung cancer services [14] and mental health services [15]. This is the first evidence of its use within maternity care.

### Context

The UK National Institute of Health and Care Excellence (NICE), in its practice recommendations in 2004 [16], stated that when a woman requested Caesarean section in the absence of an identified medical reason the request should be explored, discussed and recorded but that, while an individual clinician had the right to decline such a request, the women’s decision should be respected and she should be offered referral for a second opinion. The recent update of the Caesarean section Guideline [17] has continued the controversy by stating more strongly that, for women requesting Caesarean section, if after discussion and offer of support, a vaginal birth is still not an acceptable option, she should be offered one and that an obstetrician unwilling to perform a Caesarean section should refer the women to an obstetrician who will. The current recommendation also states that the overall risks and benefits of Caesarean section compared with vaginal birth should be discussed, but the evidence available upon which to base a decision is very low quality, includes only relatively short term outcomes and does not include the risks to future fertility or further pregnancies or the health of the baby.

Since publication of the recent update of the Caesarean section Guideline in 2011, there is a desire to explore more fully the experiences and opinions of both women and health care professionals involved at Birmingham Women’s NHS Foundation Trust (BWNFT). This has come from a desire to improve the pathway for all those involved and from the belief that numbers of women requesting Caesarean section following the update had risen. The Maternity and Child Health team of the West Midlands Collaboration for Leadership in Applied Health Research and Care (CLAHRC) programme is based at the University of Birmingham and undertake research in close partnership with local health services, with the aim of improving services and outcomes for patients within five years (though often much sooner than this).

## Methods

### Objective

This article documents an experience-based co-design project that was undertaken as collaboration between Birmingham Women’s NHS Foundation Trust, the University of Birmingham and women who had used the BWNFT service.

### Study design

**Fig. 1:**
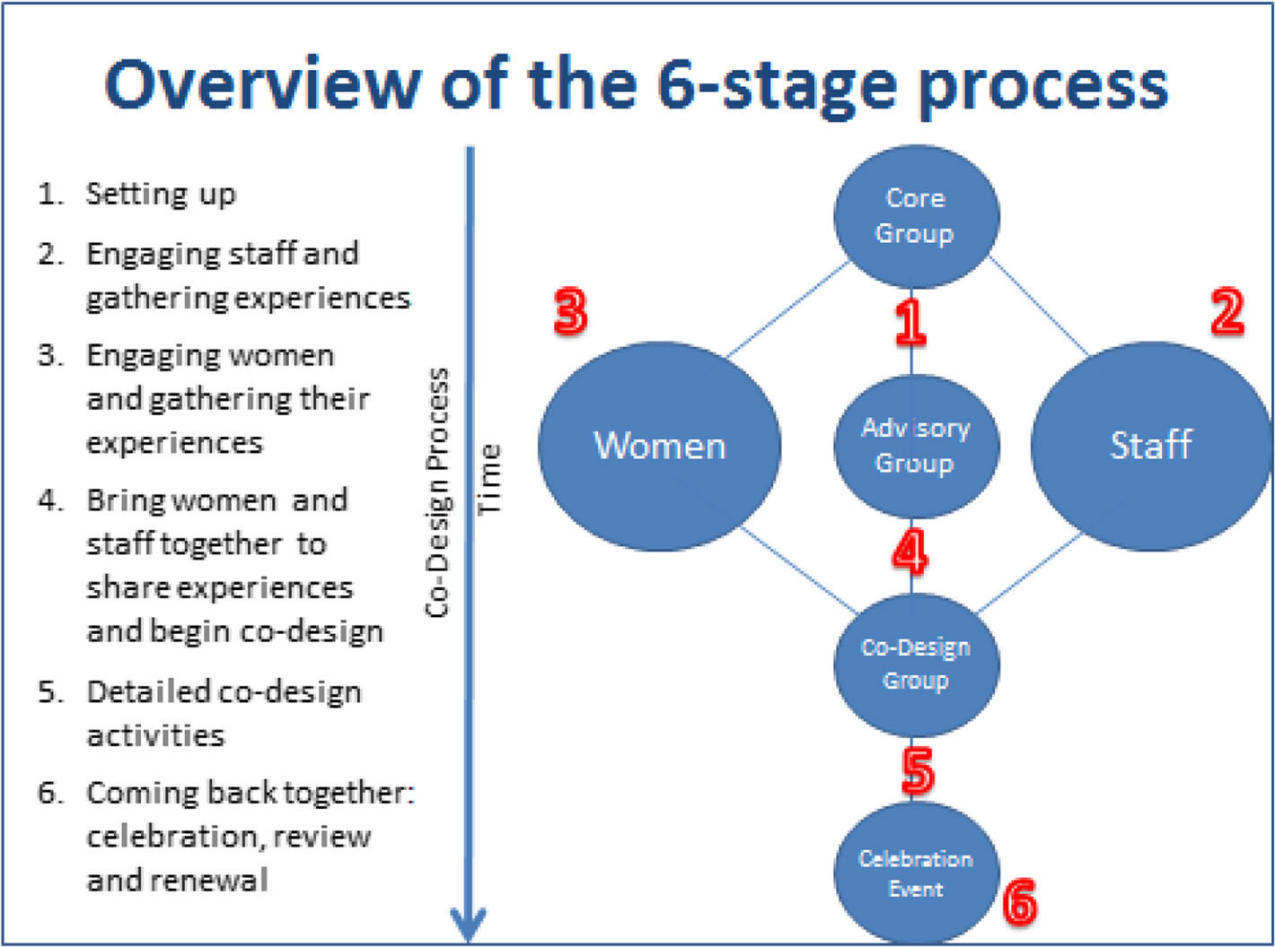
An overview of this six stage process

Experience-based co-design methodology uses service user and clinicians experiences collected using qualitative methods to jointly re-design services. Our approach to was formed by the free to access online toolkit published in August 2012, incorporating several case studies which were developed through collaboration between quality improvement practitioners and academics, and disseminated through the King’s Fund charity (http://www.kingsfund.org.uk/projects/ebcd). This gave detail in 16 sections to the process which involved eliciting the views of health care professionals and patients to ‘co-design’ service improvement. The toolkit suggests using non participant observation and semi structured qualitative interviews with staff and patient with separate workshops to discuss findings. Film clips are used to demonstrate the views of patients along the care pathway at a joint workshop, following which small co-design groups to work on different issues and a final celebration/review event is held. An overview of the experience based co-design process is presented in Fig. 1.

### Research team and study oversight

The core team consisted of SK (researcher with a clinical midwifery background), NG (sociologist and methodological specialist), SD (research fellow), NJ (obstetrician) and RH (service user/mother) offering a range of perspectives on the project. In addition to this, the project advisory group consisted of another obstetrician, another service user and two midwives. Permission for the study was obtained from the NRES Committee West Midlands - Black County (12/WM/0270). The study took place between January and December 2013 and was sponsored by BWNFT and permission was also obtained from their Research and Development Department.

### Participant selection

The experiences of both clinical staff and women who had experienced the maternal request for Caesarean section pathway were sought. Health care professionals included a sample of community midwives (who see women in the antenatal period), the Consultant Midwife (who sees women during their decision process for maternal request for Caesarean section and consultant obstetricians (who have to agree to the request for Caesarean section). Consultant obstetricians and the Consultant Midwife were identified by NJ (the lead obstetrician for Delivery Suite). The Community Midwifery Manager identified a sample of community midwives through the individual team leaders. Women were identified using the electronic systems at BWNFT by NJ and were sent a letter informing them about the study and asking them to contact the University team if they were prepared to be interviewed about their experiences. Exclusion criteria were women who gave birth by any other mode, women under 16 years old or women who were not fluent in spoken English.

Information about the study was sent to all those identified, and if a reply slip was returned following one reminder, responders were contacted to arrange an interview. All participants gave informed consent. Once a reply slip had been received from women we contacted NJ to find out whether or not the woman concerned had a clinical indication for Caesarean section. This enabled us to purposely/purposefully sample women who had, and did not have, a clinical indication, and enabled us to explore any differences between the two groups. Once participants had responded to the letter, SD telephoned them to discuss the project further and to arrange an interview if they agreed.

The Research and Development Department of BWNFT collected women’s baseline characteristics (age, ethnicity, parity, whether they speak English and postcode). The researchers informed them of those who responded and they provided the characteristics listed above for the responders and non-responders. This enabled us to explore differences between the groups.

### Setting

Interviews for clinical staff were undertaken at their place of work. Interviews for women took place at their home to facilitate women to be able to open up and talk freely in a safe environment. The women’s, staff and joint workshops were all held in meeting rooms in the BWNFT.

### Data collection

Due to limited time and resources, we opted to focus on collecting interview data from staff and women using the services. Members of the project team and advisory group were familiar with the services, through working there or having used the service, and we used their experience in lieu of non-participant observation to help orientate ourselves to the service. This is a common adaptation of the Experience-Based Co-Design approach [18].

SD conducted all the interviews and took written consent from participants before interviews commenced. Interviews were digitally audio recorded, rather than video recorded as recommended by the Kings Fund guidance, for reasons of resource and of increased anonymity for participants. Again this is a common adaptation of the Experience-Based Co-Design method [18]. The interviews were semi-structured using a topic guide with broad topic areas (background, experience of pregnancy, choice to have a Caesarean section, views on risks and benefits of Caesarean section, interaction with health care professionals, interactions with family and peers, experience of antenatal and postnatal care, reflections and future plans) but the emphasis was to elicit individuals’ own perspectives freely. The topic guide was developed from a literature review, discussions within the project team and refined as necessary during the first few interviews.

The interviews with women aimed to explore women’s experiences of requesting a Caesarean section, with and without clinical indications, and to discuss the reasons for that decision and their experiences of the health care systems in place currently. The interviews were able to explore in more depth the reason for their decision, how health care professionals responded to their request, whether this affected their antenatal experiences and those since birth and their bonding experiences.

During the interviews the health care professionals were encouraged to discuss their thoughts and feelings around Caesarean section for maternal request, with and without medical indications, how they managed such cases in practice, and included discussion of any perceived changes as a result of the recently issued NICE guidance.

### Data analysis

The audio-recorded interviews were transcribed by a recognised professional transcription service. Transcripts were reviewed for accuracy and were anonymised by the research team before analysis. The transcripts were read and coded independently by two of the researchers (NG and SD) and the emerging themes were discussed in team meetings. N-Vivo software was used to manage the dataset and the Framework method was used to managed and analyse the qualitative data, which involves comparing data across and within cases [19]. ‘Touchpoints’ were identified: ‘the key moments and places … where people come into contact with the services and where their subjective experience is shaped, and therefore where the desired emotional and sensory connection needs to be established’ [12]. These are a key part of the experience-based co-design process as they are key moments or events that stand out for those involved as crucial to the women’s experience of care and are used to help inform and structure the co-design meetings. The process of identifying touchpoints was undertaken collaboratively by the core team who read a selection of transcripts independently to identify the key touchpoints. These were compared and discussed in the Advisory Group meeting to ensure consistency of approach.

Use of an established method (in this instance the Framework method [19]) of analysing the interview data increases the rigour of finding. Ensuring the reliability and face validity of findings, through feeding back at various stages to participants and inviting comment, is integral within the co-design process. Findings from the in-depth interviews are corroborated by the individual group workshops, and a summary sent to all those interviewed (if they could not attend the workshop) for comment. Overall, processes and results were guided and agreed at each stage by the multidisciplinary Advisory Group with adoption by the Maternity Trust of the new pathway (through the Trust routine processes) ensuring findings were seen as validated.

### The co-design process

Invitations were sent to all the women who had been interviewed to participate in the workshops. For the health care professionals, all relevant workshops were widely advertised within the Trust to encourage not just staff who had agreed to be interviewed to attend.

First, workshops were held with the women and the health care professionals separately so the findings could be shared and to ensure these resonated with the separate groups. In the health care professionals’ workshop, data were presented to the participants and discussions included topics such as the issues of working with women and professional differences of opinion. In the women’s workshop, data from the women’s interviews were presented and the group agreed the key ‘touchpoints’ along the pathway that would be presented at the joint meeting. Each group also identified their top three priority areas for service improvement.

A joint workshop was then held during which women and health care professionals worked together to make plans for the redesign of services. The workshop was facilitated by the researchers (SK, NG and SD). The workshop opened with a brief introduction to the project by SK. Audio clips from the women’s interviews were used to illustrate each touchpoint (additional written consent was obtained from the women concerned to use audio clips of them in the joint workshop). Following the workshop the group had an open and frank discussion about their responses to the audio tape. Then the group split into smaller working groups (each had at least one service user, one midwife and one obstetrician) to explore potential solutions to the problems identified. Action points were agreed and these were undertaken over the next couple of months.

The group reconvened at a celebration event, which was also attended by the chief executive of the hospital. The following were agreed: changes to the pathway, a new leaflet for women regarding possible mode of birth, and changes to the leaflet for women having elective Caesarean section. Plans for both the short and longer term were also agreed.

### Storage of data

Digital recordings were stored in an electronic file, which only the research team had access to. Only those required to transcribe the recordings listened to them. Once transcribed and checked for accuracy the digital files were destroyed. In line with current practice the transcripts will be stored for 15 years. All data will be stored and archived in line with the BWNFT policies.

## Results

### Participants

In total, 70 women were identified from the electronic systems at BWNFT for an 18 month period and were sent information about the study, and 27 women responded. Three did not wish to be involved, 24 responded that they were happy to be interviewed, and interviews were actually undertaken with 15 women (nine women interviewed had a medical indication for the Caesarean section and six without a medical indication).

The baseline characteristics of the women interviewed shows they were more likely to have had a second baby, be European and have a professional occupation than those who were invited to be interviewed. The women invited were most commonly from the least deprived areas (based on Index of Multiple Deprivation score from their post code) but this was not the case for those interviewed, who were most commonly in the third quintile. Those interviewed were similar in age to those invited (33 years) and none required an interpreter (Table 1).

**Table 1:** Baseline characteristics of women

Baseline Characteristics	Women interviewed		All women invited	
	*n* = 15	21 %	*n* = 70	100 %
Parity				
First baby	0	0 %	13	19 %
Second baby or higher	10	67 %	37	53 %
Unknown	5	33 %	20	29 %
Maternal age at CS (years) – median (std)	33 (5.54)		33 (5.22)	
Ethnicity				
Africa	0	0 %	2	3 %
Asia	0	0 %	11	16 %
Caribbean	0	0 %	2	3 %
European	13	87 %	49	70 %
Other	2	13 %	6	9 %
Index of multiple deprivation quintile				
1	2	13 %	22	31 %
2	3	20 %	15	21 %
3	6	40 %	18	26 %
4	1	7 %	7	10 %
5	3	20 %	8	11 %
Occupational classification				
1 - Managers and Senior Officials	1	7 %	5	7 %
2 - Professional Occupations	4	27 %	12	17 %
3 - Associate Professional and Technical Occupations	1	7 %	6	9 %
4 - Administrative and Secretarial Occupations	1	7 %	4	6 %
5 - Skilled Trades Occupations	0	0 %	1	1 %
6 - Personal Service Occupations	0	0 %	1	1 %
7 - Sales and Customer Service Occupations	0	0 %	1	1 %
8 - Process, Plant and Machine Operatives	0	0 %	0	0 %
9 - Elementary Occupations	1	7 %	1	1 %
Not in formal employment	0	0 %	2	3 %
Unknown	7	47 %	37	53 %
Interpreter required for mother				
Yes	0	0 %	0	0 %
No	15	100 %	70	100 %
Reason for Caesarean section				
Medical indication	9	60 %	35	50 %
No medical indication	6	40 %	35	50 %

Of the health care professionals, 19 obstetricians at BWNFT were sent information about the study, 14 responded that they were happy to be interviewed, and 10 obstetricians were actually interviewed. Of those interviewed the average age was 46.3 years. Six were Obstetricians and four were Obstetrician/Gynaecologists, five male and five female and all had over 15 years’ experience.

Interviews were undertaken with 11 community midwives who responded to a request from their Team Leaders. The Consultant Midwife was also interviewed. The midwives interviewed were White British, except for one who was Iranian. Their average age was 49 years and average number of years as a midwife was eleven- this varied between two and 31 years. Eleven were Band 6 and one Band 8; one had a Diploma, 10 a Degree and one a Masters. All the midwives were female.

### Health care professionals workshop

There were 17 participants with a mix of community and hospital midwives, midwifery managers, student and research midwives (15 in total) as well as obstetric consultants (two). A description of the study was given, followed by a summary of the findings from the health care professionals’ interviews.

**Fig. 2:**
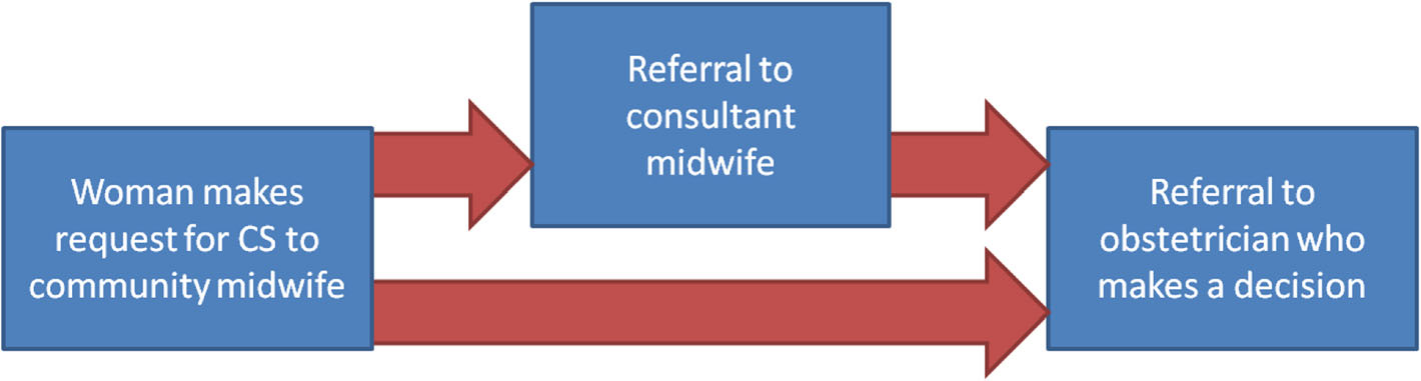
Pathway for women requesting CS as described by healthcare professionals

The pathway for women requesting a Caesarean section was seen as relatively simple when described by staff, as shown in Fig. 2.

Discussions included how staff values and experiences influenced the pathway for women and how these issues could be changed to improve the current service. Three issues in the pathway were identified by the group as a priority for discussing in the joint workshop:

#### Information provided to women

The interviews identified variation in verbal information given to women (alongside the standard leaflets):

*“I think for women, unfortunately, it’s a case of who you see” (O4)*

Some of the health care professionals spoke about managing and concealing their personal feelings and opinions about maternal requests for Caesarean section.

*“You can have your own thoughts in your head, and at the back of my head I might be thinking ‘Oh my God, you want a section just because it fits in with your life, how selfish!’ but I’m not going to put that across to the lady. I would still talk about it and not laugh her off and still give her the same pathway as I would for anybody” (M5)*

It was agreed that the discussion should include quality of information, inconsistency of information, lack of research based information, and the bias of health care professionals in what information is shared.

#### Timing of the discussion about mode of birth

The health care professionals also spoke about the importance of timing in relation to when to talk to women about their choices and options.

*“Some women will say it as soon as they walk in through the door. So at the first visit, ‘I’m not having a vaginal birth.’ Other women won’t say anything and they worry, worry, worry, and then they’ll say this two weeks before their due date, and then you’ve got no time to work with them to try and sort it out. They are by far and away the most difficult ones. The ones that come in right at the start and say, ‘I’m not happy about a vaginal birth, I want a section’ then you’ve then got the rest of the pregnancy to be able to work with them. But it’s very variable when they bring it up” (O4)*

This felt frustrating to health care professionals in terms of the extent to which they were able to provide good care:

*“We usually go through … Are you happy with what’s happening? Are you happy with where you’ve booked your delivery? Some of them feel they can’t discuss home delivery at booking because they think we are against home deliveries. And you get to the birth plan talk and she’ll suddenly say ‘I’m hoping on a home delivery’ and I’m like ‘Why didn’t you say that in the beginning?’ Do you know what I mean? You’ll get to the 36 weeks talk and it’s all about ‘I want a Caesarean section’, so why didn’t you say that in the beginning” (M1)*

It was agreed that the timing of discussion and information giving for women should be discussed at the joint workshop. This included inconsistency of timing of information, when discussion is begun in late pregnancy, the rush to make suitable preparation for birth and the need for early discussion and so time to plan for appropriate referrals where necessary and for birth.

#### The role of the consultant midwife

The role of the Consultant Midwife was discussed and the part she played in the process explored. She was seen as the centre of the system as obstetricians refer onto her when a woman requested a Caesarean section:

*“The doctor says, ‘Okay, you can have a caesarean section, but you’ve got to go and see [the Consultant Midwife] first.’ It’s nothing more than a tick box exercise. The patient knows that as long as they sit there quietly, and not upset [the Consultant Midwife] and just listen to what she’s got to say, that they’re going to be able to come back and have their elective caesarean section. So we may as well not have wasted [the Consultant Midwife’s] time” (O8)*

It is, unsurprisingly, a stressful role, as the Consultant Midwife herself explained:

*“I felt very much that I had been if you like caught out in the middle … she then got her Caesarean section and it was almost like “oh there, there, sorry I made you go through that, I’ll do your section for you”, and actually I felt unsupported and quite vulnerable … having if you like, explored it what I considered to be appropriately with her, trying to get her to explain her fears so I can try and help manage them”*

It was agreed that this would be discussed at the joint workshop and would include the inconsistent and complex process for women requesting Caesarean section without medical indication, the central role of Consultant Midwife in the pathway for women and the conflicting role of Consultant Midwife (persuading women to change their minds and try for a vaginal birth vs the need to support women in their choice).

Other themes that emerged from the discussion included the way that a woman’s decision to have Caesarean section was recorded, the need for women to repeat reason for the Caesarean section request to each health care professionals who cares for them and the lack of detailed recording of discussion between women and health care professionals when requesting Caesarean section.

### Women’s workshop

There were three women and four researchers present at the women’s workshop.

**Fig. 3:**
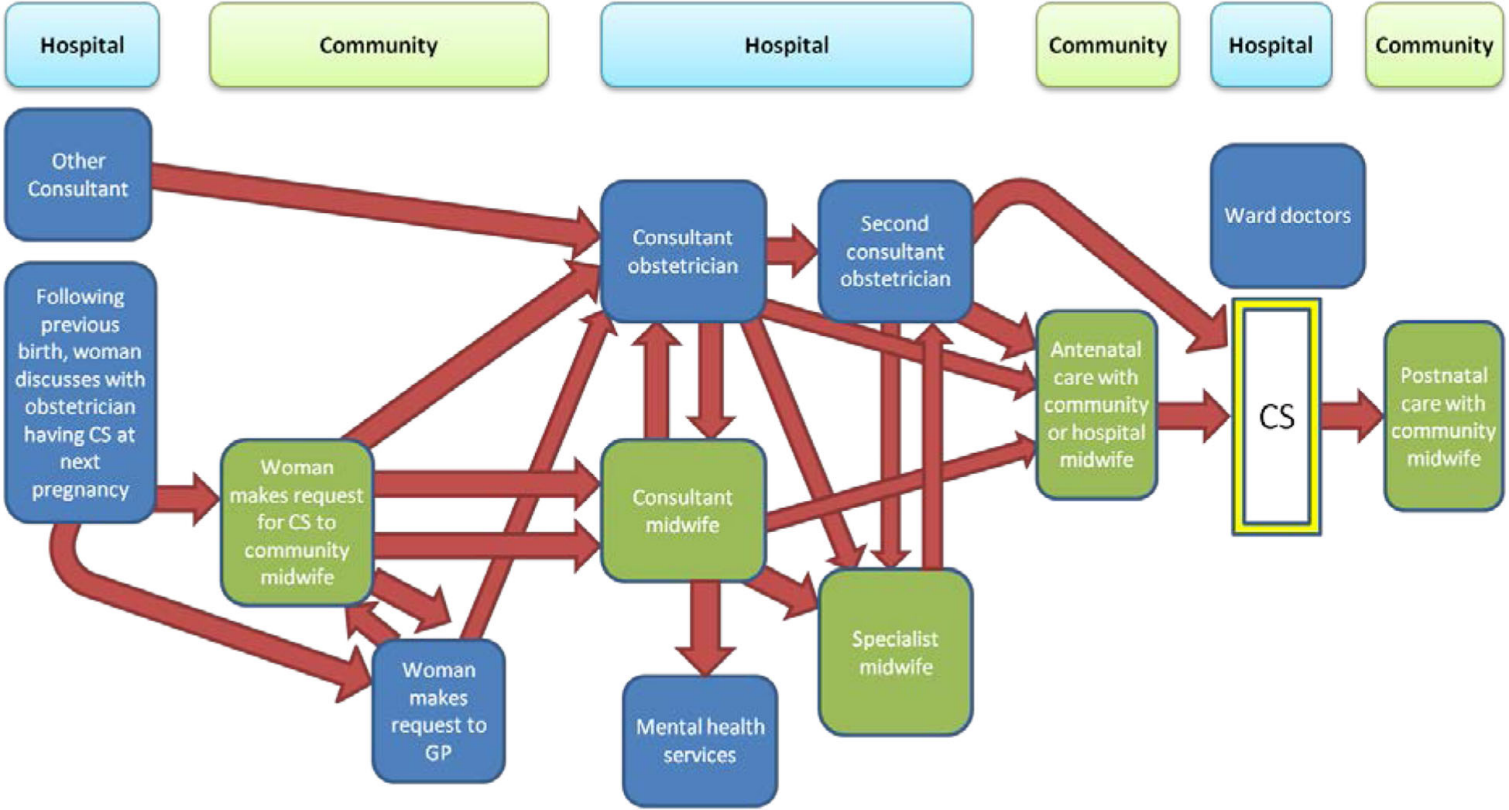
Pathway for women requesting Caesarean section as described by the women interviewed

As a result of the 15 interviews conducted with women who had requested Caesarean section, the current pathway that women had experienced in practice was documented and is illustrated in Fig. 3. This pathway was used as a basis for discussion of the key touchpoints by the women in this workshop.

The women identified three priorities for discussion at the joint workshop:

#### Information available to them

The women felt that good quality information on the risks and practicalities was missing and this included both short and long-term risks and benefits of elective Caesarean section (written information was related to Caesarean section generally and not specifically for women requesting caesarean and verbal information from midwives and consultants varied), information on the internet, and practical information given in advance of the operation.

It was also agreed that there was a lack of information about what the Caesarean section surgery experience is like and of the risk information comparing vaginal birth, emergency and elective Caesarean section and that these areas.

#### Delayed decision making

The effect a delayed decision had on experience of pregnancy was to cause unnecessary anxiety:

*“the impression you get from the midwives, that normal delivery is the best thing for everyone … ‘Oh, we can talk about that later. We can talk about that later.’ And I think if it’s, you know, I think when you’re pregnant the end, like, last bit is, kind of, praying on your mind from the moment you find out about it, really” (W1)*

#### The need to repeatedly defend their decision to have a Caesarean section

Women felt they had to continually repeat and defend their decision to each different healthcare professional they saw and that ‘no-one was listening’. They also felt that long term risk information was used to ‘ram home risks’.

*“I had a very traumatic meeting and I was made to feel like it was the worst decision I could possibly make … I’d been very aware of the 50,000 list of risks and the one positive … I kept going back to the same consultant who kept trying to talk me out of it. In the end we had to be firm in our decision” (W6)*

Women felt that those health care professionals were, at all stages, judging or stereotyping them, when in fact their decision was a carefully thought through, and sometimes very difficult, decision.

*“This midwife was lovely until I said I was having a* Caesarean section *… a complete attitude change … I explained to her the reasons and she was very dismissive from that point” (W6)*

### Joint Workshop

This was attended by 15 people (five women, two obstetricians, four midwives and four researchers). A presentation was given describing the study to date and a pathway for women was demonstrated using audio clips to describe the ‘touch points’ as agreed at the women’s workshop. Additional consent was obtained from the women for use of audio clips from their interviews. The following touchpoints were identified:

### Making the request to the community midwife

Some women found that discussing their request for Caesarean section with their community midwife could be a difficult experience. In these cases they found there was little clarity on the process, little information given to them to help make the decision and some felt that their midwife was judging them for their decision, which compromised the relationship.

*“I mean I didn’t see, unfortunately I didn’t see the same midwife, it was a different one every single time, so the midwives that I went to obviously check your blood pressure and your weight and things like that, they were “your decision”. They all said it was your decision, your decision, your decision. And then obviously when I went to the clinic they said “Oh it’s the consul- obviously it’s the consultant’s decision to make that”. (W2)*

### Making the request to the consultant obstetrician

If a woman was given an appointment with one of the BWNFT consultants who was not personally supportive of maternal request for Caesarean section, the experience could be frustrating and distressing. The women often had to ask for a second opinion.

*“When I had my 12 week scan appointment I’d got a consultant appointment at the same time and I’m pretty sure I mentioned it there and then and he was having none of it. He really brushed it aside, made me feel like it was quite a silly request and I came away feeling frustrated because I’m a 30 plus year old woman, I know my own mind, I’m not silly about unnecessary medical procedures and he made me feel very small. (W12)*

### Making the request to the consultant midwife

If women were sent to discuss their decision with the Consultant Midwife when they already sure that they had made the right decision, they could find the consultation unnecessary, and sometimes upsetting if they felt that they were being pressurized into changing their mind, rather than the supportive process that was intended.

*W12: “It was fine in itself, she was a perfectly nice lady, I just knew it was something that I had to do to get to the next step in the process of them saying yes to the elective section. So it was kind of like we talked for an hour but to me there was no point, she wasn’t going to say anything that made me change my mind. It was interesting in that when we went through, because I myself got all of my medical notes, I paid for them so I could just read everything because obviously it traumatised me quite a lot and I needed to see everything especially when I found out I was pregnant the third time. And she did go through that and find a few things that I hadn’t either found or didn’t understand because of the medical jargon so from that aspect that was useful. There were certain things that the doctors did to try and stop the bleeding that I’d got questions about and she answered those so I suppose yeah looking back it was useful in some ways but it didn’t make any difference to my decision”.*

### Mental health services

Only one participant was referred to the mental health services and she felt that it was an inappropriate referral.

*“It was quite difficult, my partner was there and there were a lot of questions about everything, going back to my childhood, whether my parents are divorced, remarried, have children of their own, which I’m not really sure how that comes into me deciding whether I want a C-section or not. So I found that quite difficult just because it was probing into my life in general and even though my husband has known my family for years and knows everything, to have to answer the questions it felt very difficult. And at the end of it she said that she couldn’t support my request on medical grounds so basically I wasn’t, it sounds awful but basically I wasn’t crazy, there are no mental health issues, it was more anxiety based. (W12)*

### Antenatal care

During the antenatal care the women received, they found that they had to repeatedly come out as having requested a Caesarean section and felt that they were required to defend that decision repeatedly. This

*W4: “I think it added to the stress. I think the, kind of, what seemed to be always questioning, and this, kind of, whole laying it on thick, but, you know, the repetitiveness about the risks and the problems and the major abdominal surgery, just adds to the stress [okay], you know. And I didn’t really feel I did have a choice, or, when I say I didn’t feel I had a choice, I felt it, you know, I really needed to have a caesarean, because the benefits outweighed the risks, really”.*

### Postnatal care in the community

The feelings of being judged for their decision continued after the birth sometimes in social situations, but also in interactions with health care professionals.

*“One of the generic checks when they’re a certain age, I don’t know if it was 8 week check or 12 week check or something and I took him to the doctors and the doctor was asking about his birth and I said it was an elective and he said oh I’m surprised they let you get away with that. And that really, really annoyed me so I’ll never see that particular doctor again and I just thought I don’t know how you can make a judgement when you have no clue about like my previous history and nobody just let me get away with anything, I had to fight tooth and nail to do it. So yeah, but that’s the only negative comment that I’ve had”.(W12)*

There was then discussion of the topics prioritised by the individual health care professionals and Women’s group which were strikingly similar and are detailed in Table 2.

**Table 2:** Agreed priorities taken to joint workshop from health care professionals and Women

Priorities taken to joint workshop from health care professionals	Priorities taken to joint workshop from women
1. Quality of information for women	1. Agreement of Caesarean section decision:
o Inconsistency of information	o A clear agreement to be made between health care professionals and women about the decision for a Caesarean section
o Lack of research based information	o Decision for Caesarean section to be made earlier in pregnancy
o Bias of health care professionals in what information is shared	o Flexibility around the decision, an opportunity to change mind at any point
2. Timing and discussion and information giving	2. Repetition of Caesarean section request and referrals:
o Inconsistency of timing of information	o Repeated discussion of Caesarean section request with health care professionals
o When discussion is begun in late pregnancy, the rush to make suitable preparation for delivery	o Multiple referrals and subsequent repetition of request
o Need for early discussion and so time to plan for appropriate referrals where necessary and for delivery	
3. Referrals and role of consultant midwife o Inconsistent and complex process for	3. Information about Caesarean section: o Lack of information about what the
o women requesting caesarean section without medical indication	o Caesarean section surgery experience is like
o Central role of consultant midwife in the pathway for women requesting CS	o Lack of risk information comparing vaginal birth, emergency and elective Caesarean section
o Consultant midwife conflicting role: persuading women to change their minds and try for a vaginal delivery vs the need to support women in their choice	
4. Recording decision – repetition of request from women	
o Women requesting Caesarean section often need to repeat their reason for the request to each HCP who cares for them	
o There appears to be a lack of detailed recording of discussion between women and health care professionals when requesting Caesarean section	

After the pathway was shared with illustrative audio clips playing at each touchpoint, there was an open discussion of responses and reactions and some of the staff were quite shocked and moved by the experiences of the women. This produced a feeling of commitment in the room about making changes and three small co-design groups (each with at least one woman, one midwife, one obstetrician and one researcher) were then formed to discuss the following prioritized topics:

- When the decision should be made that a Caesarean section was the agreed mode of birth and how that would be recorded
- How the pathway for women might be revised to reduce number and variation in the number of referrals
- What information should be given to women and when

These topics were discussed within the multidisciplinary groups, action points were agreed for health care professionals and researchers to undertake over the following weeks.

#### Final workshop

The results were discussed at the final workshop in December 2013, which was attended by 13 people (three women, five midwives, one obstetrician, the chief executive and three researchers). Here the revised pathway was agreed as were information leaflets regarding ‘The way that your baby may be born’ and ‘Elective or Planned Caesarean Section’ leaflets (Table 3).

**Table 3:** New pathway for women who request Caesarean Section

The new pathway is as follows;	
• At booking-leaflet titled ‘Information about the way your baby may be born’ given to woman with information that there will be a discussion about mode of birth around 16 week appointment • At 16 weeks - Community Midwife discusses type of birth the woman is considering*; If woman requests caesarean section*: o Assess and consider individual to see whether an appointment with health care professionals for support and information would be useful (e.g., Consultant Midwife, counsellor) Examples include previous traumatic or difficult birth, de-brief, anxious/tocophobia, undecided. Following that consultation if Caesarean section requested refer to Consultant Obstetrician. o If woman has decided on Caesarean section, make referral for consultant obstetrician appointment at 20/40 • At 20 week appointment with consultant obstetrician-detailed discussion re mode of birth: o Risks and benefits explained o Detailed documentation of discussion and current preferences o Book appointment for 24–28 • At 24–28 weeks appointment with same obstetrician o Make decision / agreement / consent about type of birth and document clearly in hospital and hand held records the final decision for type of birth that is planned (possible use of sticker or proforma in casenotes) o Discuss plans for what happens if woman goes into spontaneous labour prior to date for elective Caesarean section, including differing risks and benefits depending on stage in labour and emergency vs elective Caesarean section o Give *Elective Caesarean section leaflet* • At every subsequent antenatal appointment re-confirm (not challenge) decision (e.g., ‘Are you happy with the plan made?’) which provides opportunity for woman to change her mind but not to be repeated challenged about her decision. If booked for Caesarean section and changes her mind an appropriate plan for birth will be made dependent on individual circumstances • If previously midwife led care, then woman will remain under shared care but all other appointments (except 36/40) can be in the community • Sign consent form (if not already signed) at 36/40 and book Caesarean section for 39/40	ᅟ

Changes to the pathway for women included

- ‘Information about the way your baby may be born’ given to woman with information that there will be a discussion about mode of birth around 16 week appointment
- Not all women being referred to the Consultant Midwife but only those for whom support and information would be useful
- Seeing consultant obstetrician at 20 weeks for discussion of the risks and benefits
- Decision made 24-28weeks and recorded in notes
- Re-confirmation rather than challenging decision at subsequent antenatal appointments

At the meeting plans were made to take forward the ideas for interactive BWNFT website information on Caesarean section and to add BWNFT photos, Qs and As, possible short-term link to YouTube (visualise theatre/ experience of Caesarean section) and longer term plan to video going to theatre but these have not currently been taken forward. We also intended to submit grant proposal for detailed leaflet outlining comparison of short and long term risks for all types of births but could not identify a suitable funding source. Such a leaflet has recently been published by the Royal College of Obstetricians and Gynaecologists (https://www.rcog.org.uk/en/patients/patient-leaflets/choosing-to-have-a-caesarean-section/).

## Conclusions and discussion

This methodology has been both challenging and rewarding to use but we are confident that it has resulted in an improved pathway for women requesting Caesarean section, together with a new leaflet discussing ‘The way your baby may be born’ and an update to the information in the leaflet for women having an elective Caesarean section. One of the most notable features of the study was the marked similarity between the issues in the pathway identified by the women and the health care professionals as a priority to deal with. This was not something that we expected and meant that topics taken forward were easy to agree.

Experience-based co-design requires commitment and engagement and can be challenging [18]. In this context, an added challenge was the professional tensions between the midwifery model of care and the medical model. Evidence suggests that while obstetricians opinions have changed over time [20], with more accepting that women should be able to make an informed choice, the views of midwives are embedded in a ‘culture of normality’, in which normal birth is promoted and valued [21] and that women choosing an elective Caesarean section is at odds with this. It made the healthcare professional workshop all the more important because it gave an opportunity for those issues to be aired and discussed, before engaging with the women’s stories and experiences. Attempts to improve the pathway for women requesting a Caesarean section are likely to come up against cultural, professional and organisational challenges. Culturally, Caesarean section occupies a tenuous position in the public psyche, being both a lifesaving operation in some cases but also being seen as ‘convenient’ and ‘easier’ than a vaginal birth [22]. The pre-existing tensions between the medical and midwifery professions around the role of medical intervention in pregnancy and birth are arguably exacerbated in the case that a medical intervention is used but without a clear medical reason. Organisationally, these women trasverse organisational boundaries during their pregnancy, in particular, between the community health system and the hospital and changes to the pathway require the coordination and commitment of both systems.

A recent review, of how co-design had been used, found it had been used in at least 57 projects in many specialities within medicine and across multiple countries [18]. The review also demonstrated that the approach has been adapted by those to have used it, with this being done on the basis that the process, as outlined in the toolkit, takes too long. Most commonly, this resulted in not undertaking non participant observation and not holding the celebration event and, while over 80 % of projects reported undertaking interviews, many dispensed with the filmed component. While using film may be challenging to those planning any future project, recent evidence has suggested that use of national archive of patient experience, rather than developing local interviews was a rigorous and cost effective alternative [23]. The strength of the approach for our purposes was that it held the experience of women (and staff) at the centre of the project and required collaboration throughout. This was a powerful approach given the controversies about maternal request for Caesarean section and professional rivalries that were embedded in the context.

There were limitations to the project, which was carried out in a single hospital and with a group of women that were only partially illustrative of the diversity of women living locally and using the service (particularly in relation to socio-economic status and ethnicity). As a result, some of the emergent themes from the interviews may only be applicable to the hospital where the project was carried out. We were not able to use to most appropriate quotes to demonstrate the ‘touch points’ in the audio tape due to the requirement by the Ethics committee that we gain explicit written informed consent for the use of these. In addition, the learning about patient experience and interprofessional discussions that came from participating in the project could only be replicated by undertaking the whole process. For instance, some staff were surprised to find out that women could sense their disapproval of their choice to have a Caesarean section even when they had tried to ‘hide’ their feelings.

However, we would argue that the experience-based principles around, for instance, ensuring mode of birth is discussed early in the pregnancy were be transferable to other contexts. The new pathway and the leaflet on mode of birth could certainly be used or adapted for other maternity care contexts. We are not alone in not fulfilling all the objectives of the project or in not undertaking a formal or systematic evaluation [18]. While the research and co-design phases of the project were underway we were leading the project and so able to keep the momentum moving forward but once it passed back to the health care professionals, who have the pressure of clinical commitments as well as the researchers competing priorities, this lost its impetus. This was compounded by staff changes. Researchers considering using Experience-Based Co-Design should not underestimate the time it takes or the multi-level support required and should build in a formal evaluation, but they should be reassured of the value of this collaborative process in agreeing changes that are acceptable to all parties.

## Abbreviations

BWNFT: Birmingham Women’s NHS Foundation Trust
CLAHRC: Collaboration for Leadership in Applied Health Research and Care
NHS: National Health Service
NICE: National Institute of Health and Care Excellence
NRES: National Research Ethics Service
